# Mu-Suppression as an Indicator of Empathic Processes in Lesbian, Gay, and Heterosexual Adults

**DOI:** 10.1007/s10508-019-01491-2

**Published:** 2019-10-10

**Authors:** Katrin T. Lübke, Charlotte Sachse, Matthias Hoenen, Bettina M. Pause

**Affiliations:** Department of Experimental Psychology, Heinrich-Heine-University Düsseldorf, Universitätsstr. 1, 40225 Düsseldorf, Germany

**Keywords:** Sexual orientation, Homosexuality, Empathy, Mu-suppression, Heterosexuality

## Abstract

Self-reported empathy differs with gender and sexual orientation. The current study investigated whether mu-suppression, reflecting brain activity especially related to state empathy, also is modulated by gender and sexual orientation. Pictures of painful and non-painful actions were presented to 20 lesbians, 20 gay men, 20 heterosexual men and 20 heterosexual women, while EEG was recorded. Individual peak frequencies of mu-activity (electrodes C3, C4) were detected within the 6–11 Hz band for each participant, and mu-suppression indices were calculated. Further, verbal indicators of state empathy (pain ratings) and compassion were assessed. Only heterosexual individuals showed the typical pattern of enhanced mu-suppression in response to painful relative to non-painful pictures. Lesbian women and gay men did not show a differential mu-response. Moreover, they felt less compassion compared to heterosexual individuals. In line with this finding, the more compassion the participants reported, the stronger the mu-suppression in response to painful relative to non-painful pictures was. Pain ratings did not vary with sexual orientation. The lesser compassion reported by lesbian women and gay men is discussed as a mediator of their non-differential mu-suppression response. It is hypothesized that this pattern might relate to gay men and lesbian women tending to perceive the anonymous depicted actors as outgroup members, hence showing less compassion and reduced mu-suppression. As empathy is often related to negative feelings (empathic stress), a clear distinction between individuals to empathize with versus individuals not to emphasize with may well be an adaptive feature in same-sex oriented individuals.

## Introduction

Empathy can be defined as “emotional and mental sensitivity to another’s state, from being affected by and sharing in this state to assessing the reasons for it and adopting another’s point of view” (de Waal & Preston, 2017, p. 498). This capacity, spanning both affective and cognitive components, might have evolved in order to foster prosocial behavior and to aid in the understanding and prediction of others’ behavior (Smith, 2006); hence, it appears crucial in almost any social interaction (not only) among humans.

Evolutionary theories proposing ideas as to why same-sex orientation (or same-sex oriented sexual behavior) has not been selected against during evolution, given its inherent low reproductive success, focus, among other traits like reduced aggression, on empathy and altruism. Miller (2000) proposed an evolutionary model featuring male same-sex orientation as a polygenic trait, sustained by a balanced polymorphism: Possessing single alleles contributing to same-sex orientation would make for heterosexual carriers being more sensitive and empathic than non-carriers, thus being attractive mates and consequently successful in reproduction. Possessing several such alleles would produce male same-sex orientation. Based on primate literature and the anthropological record, Kirkpatrick (2000) presents support for same-sex oriented sexual behavior resulting from an individual selection for reciprocal altruism, which is of fundamental importance for the formation and maintenance of same-sex alliances. These alliances have reproductive advantages such as mutual aid in resource competition and cooperative defense and are supported by same-sex oriented sexual behavior. To this end, same-sex oriented sexual behavior can be defined as a survival strategy, and not a reproductive strategy.

In general, differences between (presumably heterosexual) men and women regarding empathic processes have been studied by far more extensively than differences between lesbian women and gay men and heterosexual individuals. Research has shown that, aside from self-report, in which women typically score higher than men (e.g., see Wright & Skagerberg, 2012), women outperform men in a wide variety of empathic tasks. For example, a female advantage has been reported for emotion recognition in the visual and auditory modality, that is recognizing another’s emotion by her facial expression or the emotional tone of her voice (for recent reviews, see Christov-Moore et al., 2014; Kret & De Gelder, 2012). Moreover, it has been shown that women compared to men are more aroused and more concerned with others who are in a negative affective state (Kuypers, 2017). Men and women also differ in their neural responses to empathy-relevant information, such as observing others in pain (Han, Fan, & Mao, 2008), watching chimeric emotional faces (Rueckert & Naybar, 2008) or evaluating the emotional state of another individual (Schulte-Rüther, Markowitsch, Shah, Fink, & Piefke, 2008). Even brain responses to subtle socioemotional cues as contained in human body odor appear more pronounced in women than in men (Pause, Lübke, Laudien, & Ferstl, 2010). While gender differences in empathy have been discussed as being due to men and women simply adhering to their specific gender role, recent reviews including research in non-human animals and young children propose highly conserved biological roots for gender differences in empathy (Christov-Moore et al., 2014).

Compared to gender differences in empathy, the pattern of results is by far less consistent when lesbian women are compared to heterosexual women, and gay men compared to heterosexual men. Several studies report heightened empathic traits in lesbian women and gay men compared to heterosexual individuals: Gay men self-describe as more empathic than heterosexual men (Salais & Fischer, 1995; Sergeant, Dickins, Davies, & Griffiths, 2006), and gay men also report stronger altruistic values than heterosexual men (Cochran, Mays, Corliss, Smith, & Turner, 2009). Similarly, lesbian women report a wider range of altruistic values compared to heterosexual women (Cochran et al., 2009). However, examining emotion recognition performance, Rahman, Wilson, and Abrahams (2004) did not find any effects of sexual orientation, neither in men nor in women. In contrast, others report that lesbian women are less accurate than heterosexual women when judging a target individual’s thoughts and emotions (Ruben, Hill, & Hall, 2014). Non-heterosexual (including lesbian as well as bisexual women) women have even been shown to display a heightened systemizing (ability to analyze systems) rather than empathizing (ability to understand others’ emotions, see Baron-Cohen, 2002) cognitive style compared to heterosexual women (Nettle, 2007), a pattern typically found in heterosexual men. The only brain imaging study focusing sexual orientation-related differences in central nervous activation during an empathizing task reported that heterosexual women and gay men compared to lesbians and heterosexual men showed more activation in brain areas involved in inferring the mental states of others (Perry, Walder, Hendler, & Shamay-Tsoory, 2013). The overall inconsistencies when comparing empathic processes of gay men and lesbian women with those of heterosexual individuals might be related to a heightened ingroup bias in gay men and lesbian women (Cadinu, Galdi, & Maass, 2013; Fasoli et al., 2018; Simon, Glässner-Bayerl, & Stratenwerth, 1991). Empathy is much stronger when focused on ingroup members compared to members of the outgroup (for review, see Eres & Molenberghs, 2013). Thus, depending on a given design, gay men and lesbian women might have inadvertently “responded” to either ingroup or outgroup members. However, in total, both theoretical considerations and empirical data tentatively link a same-sex orientation to heightened empathic skills at least in men (although see Rahman et al., 2004), while the pattern is less clear for women.

According to the Perception–Action model of empathy (Preston & de Waal, 2002), emotional states of others are understood via embodied representations. The human mirror neuron system is crucially involved in such shared representations, as its networks are active when actions are performed or observed (Rizzolatti & Craighero, 2004), but also during action understanding (Iacoboni et al., 2005) and emotion recognition (Leslie, Johnson-Frey, & Grafton, 2004). The primary sensorimotor cortex is one key area of the mirror neuron system (Rizzolatti & Craighero, 2004), and its activation can effectively be measured using electroencephalographic (EEG) techniques. The suppression of EEG mu-activity (oscillations typically consisting of frequencies in the range of 8–13 Hz), reflecting EEG desynchronization resulting from thalamocortical stimulation, is indicative of sensorimotor cortical activation (for overview, see Arnstein, Cui, Keysers, Maurits, & Gazzola, 2011; Pineda, 2005). Several studies have reported a linear relationship between the extent of mu-suppression and the degree of self-reported empathy (Cheng et al., 2008a, 2008b; Yang, Decety, Lee, Chen, & Cheng, 2009).

A common way to study mu-suppression is by examining empathy for pain, which is an evolutionary highly significant state. While observing another individual in pain, one does not sensorily experience the feeling, but understands the distress by perspective taking (see Jackson, Meltzoff, & Decety, 2005; Singer et al., 2004). Mu-suppression has been shown to vary with emotional trait empathy (empathic concern, Hoenen, Lübke, & Pause, 2017; empathic distress, Hoenen, Lübke, & Pause, 2018). On the other hand, it is highly susceptible to top-down processing, as its magnitude depends on how much an individual is inclined or able to take over the perspective of someone else (Hoenen, Lübke, & Pause, 2015; Hoenen, Schain, & Pause, 2013; Woodruff, Martin, & Bilyk, 2011). As such, it can be considered to also reflect cognitive aspects of empathy. Mu-suppression is further affected by gender: Women show stronger mu-suppression than men during action observation (Cheng et al., 2008a; Cheng, Tzeng, Decety, Imada, & Hsieh, 2006), as well as during the observation of others in pain (Yang et al., 2009).

Given that several lines of research propose differences between gay and heterosexual men in empathic skills, and that reports on differences between lesbian and heterosexual women in empathic skills are less consistent (and less well studied), the current study aimed at systematically investigating sexual orientation (same-sex oriented vs. heterosexual) related differences in state empathy in both men and women (in line with this notion, analyses focusing androphilic vs. gynephilic individuals were not of interest in the present study). Here, the suppression of the EEG mu-rhythm during observation of others in pain was used as a sensitive neurophysiological correlate for state empathy. The participants were further asked to judge the extent of pain probably experienced by the depicted individuals, serving as a verbal indicator of state empathy. Trait empathy was measured using a German adaption of the Interpersonal Reactivity Index (IRI, Davis, 1983). In order to control for possible effects of gender role, the participants answered the German reconstruction of Bem’s Sex Role Inventory (BSRI, Bem, 1974). It was expected that sexual orientation would affect both state markers of empathy (mu-suppression, pain ratings) and trait empathy (IRI), and that gender role would not account for these differences.

## Method

### Participants

Participants were recruited via flyers distributed at the university campus and at local bars, as well as via Facebook. In order to increase the internal validity of this pilot study, a rather homogenous participant sample was selected. Of initially 118 applicants, 83 individuals met the inclusion criteria of not reporting any neurological conditions or mental disorders requiring an intake of medication, not taking any drugs, having normal or corrected to normal vision, and being right-handed. Moreover, participants were asked which of the terms “lesbian,” “gay” or “heterosexual” would best fit how they themselves would label their sexual orientation and were assigned to their respective experimental group (lesbian women, heterosexual women, gay men, heterosexual men) based on their answer. Participants whose self-chosen label of sexual orientation would not match any of these categories (e.g., individuals disclosing as bisexual or asexual) were not included into the present study. Due to recording problems (*n* = 1) and outliers within the EEG data (deviation of the mu-suppression index across C3 and C4-electrodes from *M* ± 3 SD, see EEG Procedure: Recording and Analysis, *n* = 2), three participants were excluded. As a consequence, a total of 80 participants (20 lesbians, 20 heterosexual women, 20 gay men and 20 heterosexual men) aged 18–43 years (*M* = 24.8, SD = 5.1) were included in the final sample. Right handedness was ensured using the Annett Handedness Questionnaire (Annett, 1970), and normal visual acuity (> 80%) was ascertained via Landolt rings (EN ISO 8956, Oculus, Wetzlar, Germany). In addition to disclosing as gay, lesbian or heterosexual, respectively, the participants answered the Kinsey Scales (Kinsey, Pomeroy, & Martin, 1948) on sexual behavior and erotic fantasies. All participants described their sexual behavior and their erotic fantasies as exclusively to predominantly heterosexual or same-sex oriented. Gay men and lesbian women differed from heterosexual individuals in their self-reported sexual behavior (Kinsey Scale on sexual behavior: *t*(78) = 93.34, *p* < .001; *M* = 0, SD = 0.2 for heterosexual participants; *M* = 5.9, SD = 0.4 for lesbian women and gay men), and further in their self-reported erotic fantasies (Kinsey Scale on erotic fantasies: *t*(78) = 37.35, *p* < .001; *M* = 0.5, SD = 0.6 for heterosexual participants; *M* = 5.5, SD = 0.6 for lesbian women and gay men). All participants gave written informed consent and were compensated with course credit or a fixed sum of 17.50 euros.

### Materials

#### Visual Stimuli

Out of a picture set of 128 color pictures which had been validated in various studies (e.g., see Cheng et al., 2008b; Hoenen et al., 2015; Jackson et al., 2005; Yang et al., 2009), 64 pictures of right hands in painful and non-painful actions were used in the present study. To each non-painful picture, there was a painful equivalent (e.g., having the right hand placed at the door handle versus having the right hand placed between the door and the doorframe). The picture set included several types of pain (mechanical, thermal and pressure). To avoid any gender bias, male typical actor characteristics (e.g., hair visible on the back of a hand) were removed using Adobe Photoshop CS2 9.0. All pictures were presented in a resolution of 600 × 450 pixels on a TFT monitor (resolution: 1600 × 1200 pixels). The pictures were presented using Presentation18 (Neurobehavioral Systems Inc., CA, USA) at a distance of 80 cm to the participant’s eyes, covering a visual angle of 14.3° horizontal and 10.7° vertical. In order to record baseline activity, a white cross (2.43° × 2.43° visual angle) was presented on a black screen.

#### Scales and Questionnaires

##### State Empathy

Pain ratings (“How painful was the action for the depicted individual?”) served as a verbal indicator of state empathy. These judgments were implemented via a computer-based visual analog scale ranging from “not painful” (= 0) to “very painful” (= 100).

##### Trait Empathy

A German adaptation of the Interpersonal Reactivity Index (IRI, Davis, 1983), the Saarbrueck Personality Questionnaire (SPQ, Paulus, 2009) served to measure the participants’ trait empathy. The SPQ consists of the subscales “perspective taking” (tendency to adopt the psychological viewpoint of others), representing a cognitive factor of empathy and the subscales “fantasy” (tendency to transpose oneself imaginatively in the feelings of fictional characters), “empathic concern” (sympathy and concern for unfortunate others) and “personal distress” (feelings of unease in tense interpersonal settings), representing emotional components of empathy. Each scale includes four items and ranges from 4 to 20. A sum score of all subscales except for “personal distress” represents a “general empathy” score, ranging from 12 to 60 (Paulus, 2012).

##### Gender Role

The German reconstruction (Schneider-Düker & Kohler, 1988) of Bem’s Sex Role Inventory (BSRI, Bem, 1974) was used in order to measure the participants’ gender role. The BSRI consists of adjectives reflecting character traits either socially desirable for men (masculinity scale) or for women (femininity scale). Statistical comparison of the two scale means results in a T-value depicting the participants’ gender role (“gender role score”). Values equal to or above 2.025 resemble a masculine gender role, whereas values equal to or below − 2.025 refer to a feminine gender role. Values between − 1 and 1 indicate an androgynous gender role, and values between the gender-typed and the androgynous category refer to either a feminine or a masculine tendency.

##### Convergent Validity of Pain Depictions

The convergent validity of the pain depictions was assessed via a post-study rating (*n* = 63), asking to what extent the participants felt compassion toward the depicted individuals (ranging from 0% = “not at all” to 100% = “absolutely”).

### EEG Procedure: Recording and Analysis

After the EEG-electrodes were attached, the participants were instructed to avoid eye and body movements. Then, a visualized instruction of an exemplary trial was given. Ongoing EEG was measured during two recording blocks, separated by a break of 2–5 min. In each block, all 64 pictures of right hands in painful and matching non-painful actions were presented in randomized order. Prior to the beginning of each block, a countdown from 3 to 1 (duration 3 s) was presented. Each trial started with a fixation cross shown for a random duration varying between 2.25 and 2.75 s. Following the fixation cross, a picture was presented for a random duration also varying between 2.25 and 2.75 s, resulting in a total duration (fixation cross and picture) of 5 s. In order to maintain attention and to avoid effects of fatigue, the pictures were followed by painfulness judgments in 25% of the cases. If a given painful picture was rated in a block, its non-painful pendant was also rated within the same block and vice versa. After the rating, a countdown indicated the beginning of the next trial. The mean duration of each block was *M* = 9 min (SD = 1 min).

Ongoing EEG was recorded from 15 scalp locations (Fp1, Fpz, Fp2, F3, Fz, F4, C3, Cz, C4, P3, Pz, P4, O1, Oz, O2 of the 10/10 system) with Ag/AgCl sintered electrodes using a stretch Lycra cap (Easycap GmbH, Wörthsee, Germany). Electrodes C3 and C4 were used to measure mu-activity (Hoenen, Lübke, & Pause, 2016; Hoenen et al., 2017). As the accuracy of eye movement correction via independent component analysis increases with the number of available channels, the remaining 13 electrodes served for eye movement corrections (see below). Eye movements were monitored using a supra- to suborbital montage with supraorbital electrode placed 3 cm above the right eye and inside the vertical pupil axis, and the suborbital electrode placed 1 cm below the right eye and lateral of the vertical pupil axis. The ground electrode was placed at position AFz. Data were sampled at 500 Hz with an averaged reference and low-pass filtered online at 135 Hz using a QuickAmp72 EEG System (Brain Products GmbH, Gilching, Germany).

During offline processing, data were re-referenced to averaged earlobes and filtered with a high-pass filter at 0.5 Hz (48 dB*/*oct), a low-pass filter at 40 Hz (48 dB*/*oct) and a notch filter at 50 Hz (BrainVision Analyzer 2, Brain Products GmbH, Gilching, Germany). Eye movements were corrected via an independent component analysis (Jung et al., 2000). Independent components representing eye movement artifacts were identified by inspecting the component’s spatial topography as well as their time course, which, in case of eye movement artifacts, should be similar to that of the EOG. Within the frequency domain, none of the rejected (removed) independent components showed a peak in the mu-range.

The EEG was then segmented into epochs of 1024 data points (2048 ms), beginning 200 ms after stimulus onset (fixation cross and picture, respectively) to exclude the effects of early event related potentials on the EEG occurring within the first 200 ms (Hoenen et al., 2017; Whitmarsh, Nieuwenhuis, Barendregt, & Jensen, 2011). The epochs were visually inspected in order to detect remaining artifacts, and a total of 1.8% of data were rejected. A noncomplex fast Fourier transform (FFT; frequency resolution of 0.488 Hz) with a Hanning-Window of *α* = .50 was applied to the data.

The suppression indices were computed for each data point of the frequency spectra as log-transformed ratio of the power during picture presentation relative to the power during baseline (fixation cross, Oberman et al., 2005). A log ratio of less than zero indicates a suppression of power, whereas a log ratio greater than zero indicates enhancement of power. Since research has shown that individuals differ in their individual mu-bandwidth (Bazanova, 2012), and that mu-bandwidth in general is affected by gender and sex hormone levels (Carrier, Land, Buysse, Kupfer, & Monk, 2001; Chiang, Rennie, Robinson, van Albada, & Kerr, 2011), individualized mu peak frequencies were determined. First, the overall mu-suppression was averaged and a sample-specific frequency range was determined (see Haegens, Cousijn, Wallis, Harrison, & Nobre, 2014) by testing the overall suppression indices of the frequency bins 10 (4.883–5.371 Hz) to 24 (11.719–12.207 Hz, frequency resolution = 0.488 Hz) against zero, including only those frequency bins into the mu-range that reached an alpha level of 0.001%. As a result, the frequency bins 13 (6.348–6.836 Hz) to 21 (10.254–10.742 Hz) were defined as the study-specific mu frequency range. Within this range, the frequencies showing the maximum mu-suppression were identified for each individual and the individual mu-suppression index (extending 1 Hz above and below this individualized peak frequency) was calculated (Southgate, Johnson, El Karoui, & Csibra, 2010; Southgate, Johnson, Osborne, & Csibra, 2009).

### Statistical Analysis

In order to examine the effects of gender and sexual orientation on mu-suppression as a neurophysiological marker of state empathy, a mixed-factorial 2 (gender: female, male) × 2 (sexual orientation: same-sex oriented, heterosexual) × 2 (picture type: painful, non-painful) × 2 (electrode position: C3, C4) analysis of variance (ANOVA) was calculated. Similarly, verbal indicators of state empathy (pain ratings) were subjected to a 2 (gender: female, male) × 2 (sexual orientation: same-sex oriented, heterosexual) × 2 (picture type: painful, non-painful) ANOVA. In case of significant interactions, nested effects were calculated (Page, Braver, & Kinnon, 2003). In order to account for the small sample size, results of *t*-tests utilizing bootstrapping (2000 bootstrap samples, bias corrected and accelerated Bootstrap confidence intervals, Efron & Tibshirani, 1993) are reported additionally.

In order to examine gender- and sexual orientation-related differences in trait empathy, self-reported gender role, and judgments of the pain depictions’ convergent validity, scores of the SPQ (“perspective taking,” “fantasy,” “empathic concern,” “personal distress” and “general empathy”), the BSRI (gender role score) and compassion ratings were subjected to 2 (gender: female, male) × 2 (sexual orientation: same-sex oriented, heterosexual) between-subjects ANOVAs and followed up by nested effects analysis.

Possible linear relationships between state markers of empathy on the one hand, and gender role, trait empathy and judgments of convergent validity on the other hand were explored using Pearson’s product-moment correlations. In case of mu-suppression (averaged across C3 and C4 electrodes) and pain ratings, correlations were based on difference scores (responses to painful pictures minus responses to neutral pictures), indicating the extent to which more mu-suppression was shown and higher pain ratings were reported in response to painful relative to non-painful pictures.

An alpha level of 5% was used for all statistical tests; however, given the pilot character of the current study, effects reaching the 10% level also are reported. All analyses were conducted using SPSS 23 (IBM Corp., NY).^1^

## Results

### State Empathy

#### Mu-Suppression

**Fig. 1:**
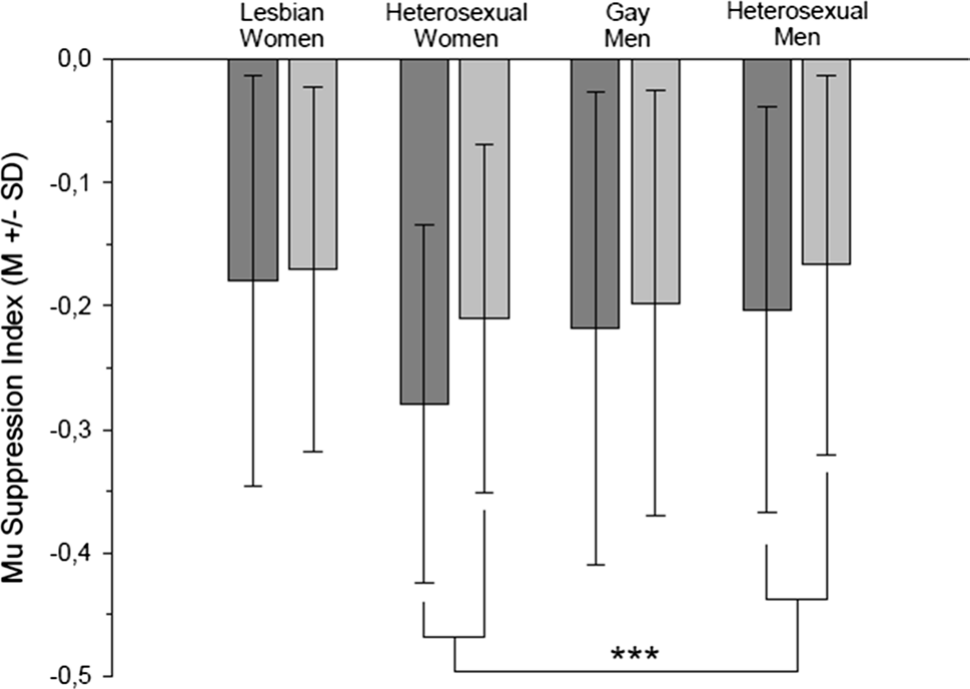
Mu-suppression index (*M* ± SD) of lesbian women, heterosexual women, gay men and heterosexual men in response to painful pictures (dark gray bar) and non-painful pictures (light gray bar). Note that more negative values refer to a greater magnitude of mu-suppression. ***Picture type within heterosexual individuals *p* < .001

In general, the participants’ mu-suppression was stronger while observing painful pictures compared to non-painful pictures (picture type: *F*(1, 76) = 13.48, *p* < .001, *η*_p_^2^ = .151). However, this effect was mainly driven by heterosexual individuals (picture type × sexual orientation, *F*(1, 76) = 3.99, *p* = .049, *η*_p_^2^ = .050, nested effects: picture type within heterosexuals, *F*(1, 76) = 16.06, *p* < .001, *η*_p_^2^ = .175, see Fig. 1). Lesbian women and gay men, in contrast, did not differ in their mu-suppression regarding the picture type (*p* = .240, *η*_p_^2^ = .018). These results were supported by bootstrapping: Heterosexual individuals display stronger mu-suppression in response to painful relative to non-painful pictures compared to lesbian women and gay men (*p* = .047, two-sided). Gender did not affect mu-suppression (*p*_s_ ≥ .184, all *η*_p_^2^ < .023).

#### Verbal Indicators of State Empathy

**Fig. 2:**
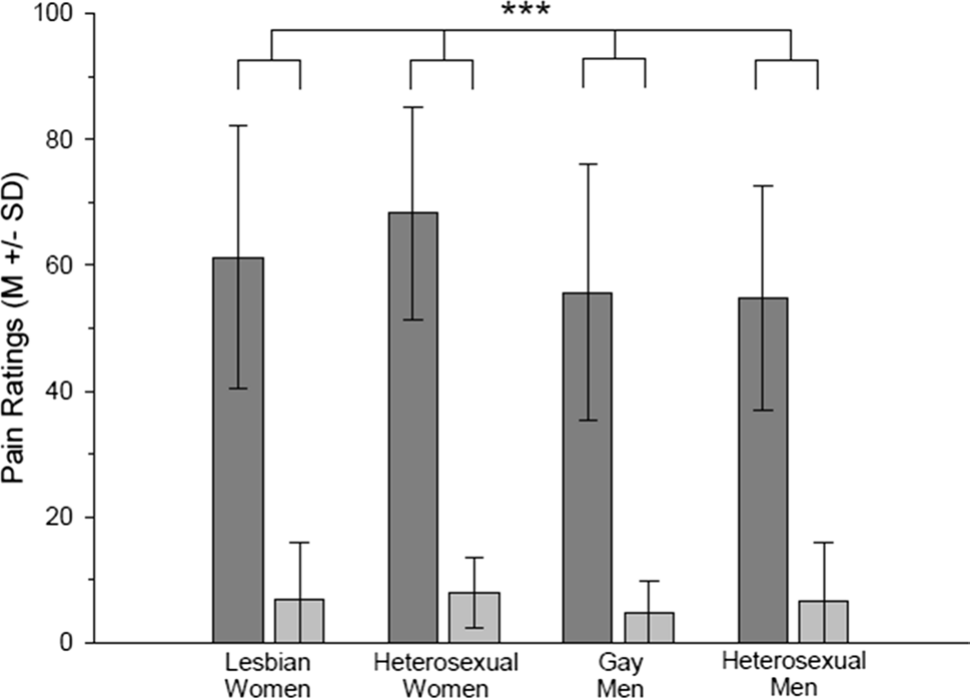
Pain ratings (*M* ± SD) of lesbian women, heterosexual women, gay men and heterosexual men regarding painful (dark gray bar) and non-painful pictures (light gray bar). ***Main effect picture type *p* < .001

Pictures of hands in painful situations were rated as more painful than pictures of hands in neutral situations (picture type: *F*(1, 76) = 612.77, *p* < .001, *η*_p_^2^ = .890). Furthermore, women judged the pictures in general as more painful compared to men (gender: *F*(1, 76) = 5.57, *p* = .021, *η*_p_^2^ = .068). This effect was mainly driven by women rating hands in painful situations as more painful than men (picture type × gender, *F*(1, 76) = 3.27, *p* = .074, *η*_p_^2^ = .041, nested effects: gender within painful pictures: *F*(1, 76) = 5.08, *p* = .027, *η*_p_^2^ = .063, see Fig. 2). Participants’ sexual orientation did not affect pain ratings (*p*_s_ ≥ .314, all *η*_p_^2^ < .013).

### Trait Empathy, Gender Role, and Validity Judgments

**Fig. 3:**
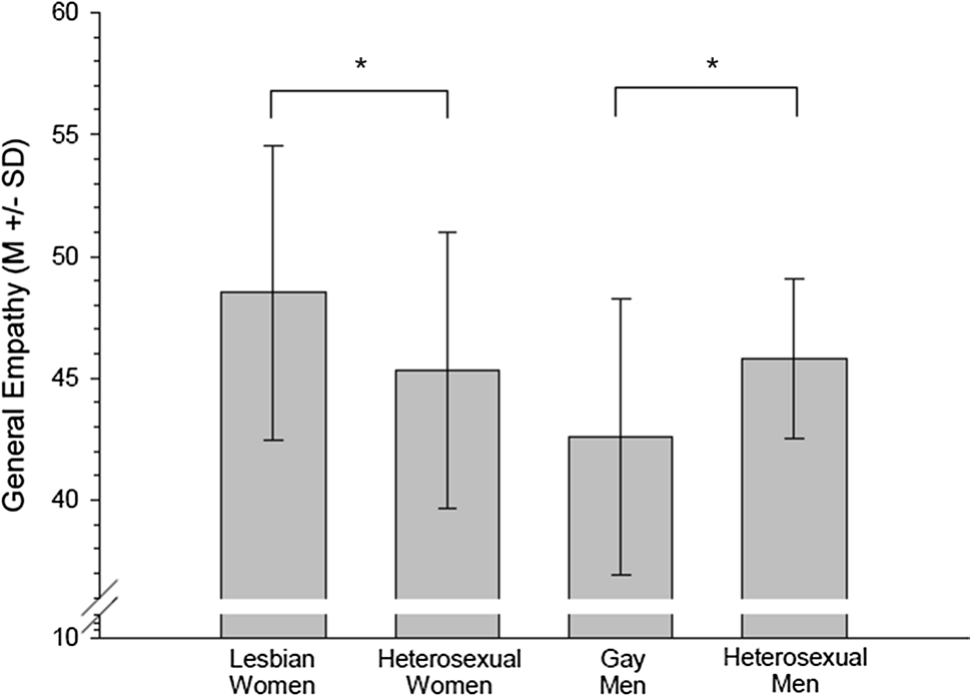
General trait empathy score (SPQ) of lesbian women, heterosexual women, gay men and heterosexual men (*M* ± SD). *Sexual orientation within women *p* = .059, sexual orientation within men: *p* = .059

As measured by means of the SPQ, women described themselves as generally more empathic than men (gender: *F*(1, 76) = 5.23, *p* = .025, *η*_p_^2^ = .064). Moreover, lesbian women reported slightly more general empathy than heterosexual women, while gay men reported slightly less general empathy than heterosexual men (gender × sexual orientation: *F*(1, 76) = 7.34, *p* = .008, *η*_p_^2^ = .088, nested effects: sexual orientation within women: *F*(1, 76) = 3.67, *p* = .059, *η*_p_^2^ = .046, sexual orientation within men: *F*(1, 76) = 3.67, *p* = .059, *η*_p_^2^ = .046, see Fig. 3).

Gay men, lesbian women and heterosexual individuals described themselves as androgynous and accordingly did not differ in their gender role score (lesbian women and gay men: *M* = − 0.49, SD = 1.71, heterosexual individuals: *M* = − 0.34, SD = 1.99, *p* = .708, *η*_p_^2^ = .002). Furthermore, women as well as men also described themselves as androgynous; however, women tended toward a more feminine (albeit still in the androgynous range) gender role description (*M* = − 0.95, SD = 1.77) than men (*M* = 0.12, SD = 1.78, gender: *F*(1, 76) = 7.07, *p* = .010, *η*_p_^2^ = .085).

**Fig. 4:**
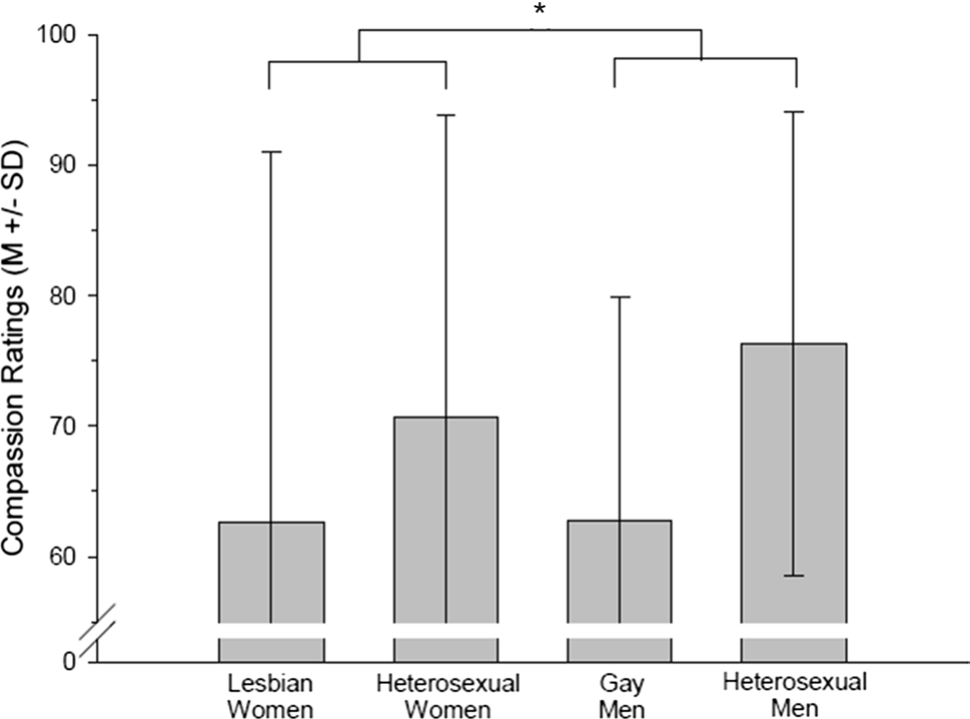
Compassion ratings of lesbian women, heterosexual women, gay men and heterosexual men (*M* ± SD). *Main effect sexual orientation *p* = .061

Out of 80 individuals taking part in the study, 63 individuals (*n* = 17 lesbian women, *n* = 16 heterosexual women, *n* = 16 gay men, *n* = 14 heterosexual men) provided information regarding the extent to which they had felt compassion toward the depicted individuals. Lesbian women and gay men felt less compassion with the depicted actors compared to heterosexuals (*F*(1, 59) = 3.65, *p* = .061, *η*_p_^2^ = .058, see Fig. 4).

### Relationships Between State Empathy and Gender Role, Trait Empathy, and Judgments of Convergent Validity

The state markers of empathy showed a significant linear relationship: More mu-suppression (more negative values) in response to painful relative to non-painful pictures was associated with higher pain ratings of painful relative to non-painful pictures (*r* = − .26, *p* = .020).

Moreover, mu-suppression was further correlated to judgments of the pain depictions’ convergent validity: The higher the participants’ self-reported compassion, the more mu-suppression (more negative values) in response to painful relative to non-painful pictures was evident (*r* = − .24, *p* = .050, *n* = 63).

Neither mu-suppression nor pain ratings were related to gender role or trait empathy (all |*r*| ≤ .16, *p*_s_ ≥ .13).

## Discussion

The current study aimed at systematically investigating sexual orientation-related differences in empathy in both men and women. The results showed that lesbian women and gay men indeed differ from heterosexual individuals in several aspects of empathy; however, the direction of these differences seems to vary with the specific empathic marker in question. In contrast to heterosexual individuals, lesbian women and gay men do not show pronounced mu-suppression during the observation of others in pain, which is considered a neurophysiological correlate of state empathy. On the other hand, lesbian women report higher trait empathy than heterosexual women, while gay men report less trait empathy than heterosexual men. Taken together, the complex picture of the current results strongly argues in favor of mediating variables affecting the relationship between sexual orientation and empathy.

In detail, although mu-suppression during observation of others in pain is evident across participants, this effect is mainly driven by heterosexual individuals. Correspondingly, gay men and lesbian women report less compassion with the depicted individuals than heterosexual individuals. Of note, sexual orientation did not affect judgments of the extent of pain probably experienced by the depicted individuals. Thus, gay men and lesbian women might indeed show less state empathy in terms of neurophysiological correlates than heterosexual individuals. At first sight, these results contradict findings from others, reporting that heterosexual women and gay men (individuals attracted to men) show greater state affective empathy than heterosexual men and lesbian women (individuals attracted to women), and also show stronger activations in brain regions involved in inferring the mental states of others (Perry et al., 2013). However, Perry et al. also reported that the effect within affective state empathy was mainly due to lesbian women featuring lower empathy compared to all others groups, and that the effects in brain activation were driven by pronounced responses of heterosexual women. This more differentiated pattern is actually quite similar to the one observed within the current study (see Fig. 1).

Further analyses showed that trait empathy, gender and gender role were unrelated to mu-suppression as neurophysiological marker of empathy. Rather, mu-suppression is related to verbal indicators of state empathy (pain ratings) on the one hand, and compassion ratings on the other hand: The higher the participants judged the painfulness of the actions depicted in painful relative to non-painful pictures, the larger the magnitude of the mu-suppression in response to painful relative to non-painful pictures. Also, the more compassion they reported, the larger the magnitude of the mu-suppression in response to painful relative to non-painful pictures was. Thus, the fact that lesbian women and gay men felt less compassion toward the depicted actors compared to heterosexual individuals might explain their non-differential mu-suppression in response to painful relative to non-painful pictures. According to de Vignemont and Singer (2006), empathic brain responses are subject to fast and implicit appraisal processes, depending, among other factors, on an individual’s relationship to the target (for discussion, see also Bernhardt & Singer, 2012). This may include any affective link to the target, but also similarity and familiarity. Hence, a target would need to be of minimum relevance to an individual in order to induce empathic brain responses. The current results might therefore be explained by lesbian women and gay men inadvertently judging the depicted actors as less relevant than heterosexual individuals. However, if lesbian women and gay men differed from heterosexuals in this relevance judgment, it is of interest why they should do so.

A possible explanation could be that lesbian women and gay men display a heightened ingroup bias compared to heterosexual individuals, and that—due to more common encounters with outgroup members than ingroup members in daily life—they involuntarily perceived the depicted actors as outgroup members. Research has indeed shown that gay compared to heterosexual men display stronger self-categorization, self-stereotyping and ingroup identification (Cadinu et al., 2013; Fasoli et al., 2018; Simon et al., 1991).

Individuals have been shown to be less responsive to outgroup members than to ingroup members in automatized action-perception related mechanisms (Molenberghs, Halasz, Mattingley, Vanman, & Cunnington, 2013), and even specifically in mu-activity (Gutsell & Inzlicht, 2010) and other aspects of affective and cognitive empathy (for review, see Eres & Molenberghs, 2013). Thus, it is likely that gay men and lesbian women differentiating to a higher degree between their ingroup and their outgroup might have resulted in a less differential mu-suppression response and in relatively weak feelings of compassion within the current study. As in general, empathy also involves negative aspects like empathic stress increasing cortisol levels (Engert, Plessow, Miller, Kirschbaum, & Singer, 2014), a clear distinction between individuals to empathize with versus individuals not to empathize with may well be an adaptive feature. Accordingly, research has shown that individuals tend to empathize less with others in pain who had previously behaved unfair compared to those who behaved fair (Singer et al., 2006). Interestingly, Perry et al. (2013), who reported heightened neural and affective markers of empathy in gay men (combined with heterosexual women), compared to heterosexual men (combined with lesbian women) presented their participants with task material explicitly featuring their respective ingroup.

In fact, the question whether the conclusions regarding gay men’s and lesbian women’s heightened ingroup bias affecting empathy indeed hold cannot be answered empirically based on the current design, which is a limitation of the current study. Future studies should therefore set out to systematically test this assumption, for example, by priming an ingroup- versus an outgroup context prior to picture presentation. Another limiting factor is the relatively small sample size on which the results are based. Given the pilot character of the current study, much effort was taken to obtain a rather homogenous sample in order to increase internal validity. Consequently, a number of applicants were not included into any of the samples due to not meeting the inclusion criteria. Results showed, however, a small but significant main effect of sexual orientation on mu-suppression, which was further supported by confirmatory data analyses (bootstrapping). The chance of having made a Type II error thus appears conceivably small, especially since effect sizes are per se not expected to be large when examining sexual orientation-related differences regarding a majority of psychological constructs.

Taken together, the current data show that the effects of sexual orientation on mu-suppression as a neural marker of empathy are not modulated by traits such as general empathy or gender role. Instead, they are linked to differences in state empathy, which in turn appear to be affected by cognitive appraisal possibly related to perceived group membership. However, further research is needed in order to clarify whether gay men’s and lesbian women’s empathic responses to ingroup versus outgroup members might indeed differ from those of heterosexual individuals.

## Footnotes

1 The datasets generated and analyzed during the current study are available from the corresponding author upon request.
